# Afterword: A Functional Analysis of the Crisis in American Society, 2020

**DOI:** 10.1007/s12108-021-09480-6

**Published:** 2021-04-02

**Authors:** Victor Lidz

**Affiliations:** Department of Psychiatry, Drexel University College of Medicine, 31 Independence Court, Chesterbrook, PA 19087 USA

## Abstract

In 2020, American society experienced a number of crises. They involved raced relations precipitated by the Black Lives Matter movement, the Covid 19 pandemic with its huge loss of life and rates of illness, the economic recession that accompanied the pandemic and efforts to control it, and the tensions of the national political campaign, followed by the refusal of President Trump to acknowledge his defeat. Each of these crises led to cleavages in the relationships of solidarity in the societal community subsystem of American society. The cleavages may prove to be the most enduring of the crises.

Larry Nichols, as editor of The American Sociologist, asked whether I would write, as an Afterword to the special issue on Talcott Parsons and Politics, a Parsonian analysis of the complex crisis in American society in the summer and fall of 2020. The present essay is a response to that request. It is not based on methodical research but only on close attention to the news as reported in the New York Times, the Washington Post, and CNN as well as commentary on MSNBC. It should be understood as an exploratory attempt to use the theory of action to analyze a complex situation, one still unfolding as I finish writing in mid-February 2021.

My effort takes two elements of Parsons’ writings as guidance. First are his macrosocial analyses addressing crises of societies: of Germany in the Nazi era, focusing on the question of how Hitler and the Nazi regime came to power; of the U.S. during and in the wake of the Joseph McCarthy movement of the early and mid-1950s, addressing the question of why allegations of disloyalty among intellectuals and high government officials stimulated a political movement with widespread support; of the U.S. in the period of the Civil Rights movement, when the nature and extent of the resulting social change remained in question; and of the U.S. during the period of student protests in the late 1960s and early 1970s concerning university authority structures and the balance in faculty efforts between research and teaching. In each of these cases, Parsons focused on the ways in which diverse social changes of previous decades had placed special stress on class and status groups that then sought new directions of change.

Second is the development of the four-function paradigm that facilitates analysis of systems of social action, including whole societies, into subsystems, an analytical schema that can be applied universally. In this case, I shall use the functional analysis of societal subsystems: economy serving the adaptive function; polity serving the goal attainment function; societal community serving the integrative function; and the fiduciary system—a collection of institutions that serve to maintain and elaborate commitments to shared culture—serving the pattern maintenance function. I will not extend this provisional analysis to the technical level of specificity that involves subsystems of the four primary subsystems of society and the symbolically mediated interchanges among subsystems of society. Rather, I use the conception of the four primary societal subsystems as general categories for organizing the analysis of complex processes affecting many aspects of contemporary American society. I will argue that the crisis focuses on institutions and relationships within the societal community, but that stresses generated in the other three subsystems of society have exerted important strains on patterns of social integration.

In his small book, *The System of Modern Societies*, Parsons argued that, in the decades following World War II, the United States had developed advanced institutions that distinguished it from all other societies, both historical and contemporary. The American economy had attained new levels of productivity, providing relative wealth and comfortable living standards for most of the population. Since the Great Depression, the political system had implemented effective policies to improve the well-being and security of citizens, notably building their confidence in the society generally. The policies had also protected the nation and its allies during World War II and then the Cold War. Basic freedoms and liberties seemed to be more broadly and securely institutionalized than in any previous period of American history. Americans took pride in having the world’s most stable and effective constitutional democracy, and the nation’s diplomacy, despite critical mistakes in Iran, Guatemala, Vietnam, and some other countries, sought to expand democracy worldwide. In the societal community, there had been a major expansion of the middle class as a proportion of the whole population. To be sure, there remained a range of distinctly ranked statuses within the broad middle class, but the proportion of citizens who experienced social recognition of “success” in their personal lives, the public respect due contributors to the general welfare, and progressively better living conditions were large as compared with pre-WWII conditions. The fiduciary system was experiencing more stable family relationships (despite a rise in divorce rates), rising participation in churches, other religious institutions, and various cultural associations, and especially increased levels of education, including higher education. New levels of educated skills were contributing to institutions of economic production, skills ranging from basic research science to engineering to corporate management to the applied professions, but also including the operation of small businesses and even the quality of assembly line work in factories.

On the international level, the U.S. seemed to be a “lead” society. It held a position of leadership at the United Nations, in a complex series of alliances, especially with the European democracies through NATO, a primary bulwark against the Soviet Union and the dependent nations of Eastern Europe, and with Japan, Asia’s lead economy. In the post-war period, the U.S. had also played a key role, although far from consistently or effectively so, in persuading European colonial powers to free their colonies while also placing them on paths toward democracy. A question to ask now is: Has loss of a lead role been a factor in the widespread feeling of malaise in American society?

## Brief Description of the Interrelated Crises

The homicide of George Floyd in police custody during the early summer of 2020 was a precipitating event that initiated public focus on deeper and longer-term troubles in American society. One focus was on the violence, and killings, that all-too-often accompanied police interaction with racial minorities, especially African Americans. Massive demonstrations arose in over 150 cities across the country in response to the police killings of African American citizens, additional instances of which came to light during the summer, notably the killing of Brianna Taylor in her home. In some cities, the Black Lives Matter demonstrations lasted several days, while in others they lasted for weeks and resulted in repeated and intense confrontations between demonstrators and police, creating an additional sense of crisis. In several cities, stores and some public facilities were vandalized and even set on fire, amplifying the sense of a crisis of social order among some members of the public.

Troubling as the police killings were, they stood as symbols of a range of other race-related problems that demonstrators discussed with journalists: discrimination throughout the justice system, continued segregation in housing, poor schooling for minority children, lack of economic opportunity, discrimination in a wide range of work settings, lack of attention to positive contributions by minority artists and intellectuals in the mass media and cultural institutions, and a lack of positive response by political leadership. In the ensuing weeks and months, attention fell on racial and ethnic injustices in practically all institutions in American society—major corporations, small businesses, the professions, universities and academic associations, the mass media, the various arts, federal, state, and local government agencies, and voluntary associations A society that had taken some pride in the changes brought about by the Civil Rights movement of the 1960s suddenly began to face the grievous shortcomings in its progress.

At the same time, the Covid 19 pandemic spread across the country and into ever larger demographic groups, emerging as a national crisis. Deaths concentrated at first in New York City and New Jersey, then spread throughout the country. Deaths mounted into the hundreds of thousands. Sickness and deaths concentrated in nursing homes, in prisons, in meat-packing plants, some other factories, and then among healthcare providers, especially nurses and nursing assistants. Hospitals in major cities and in some small towns and rural areas were overwhelmed with Covid 19 patients. Many hospitals fell into financial difficulties due to the cancellation of elective procedures.

Many businesses closed, with the hospitality industry of hotels, tourist locations, restaurants, and bars and the travel industry of airlines, Amtrak, and bus lines especially affected. Some 23 million jobs were lost over a period of mere weeks, some temporarily, others permanently, and high unemployment persisted into 2021. Many workers were left without clarity as to whether or when their places of work might reopen. Millions of small businesses closed, and many—perhaps one-fifth of all small businesses – may have closed permanently. Some large businesses, especially chain department stores, filed for bankruptcy protection. Many office workers maintained their jobs by computer connections from their homes. Many schools, colleges, and universities began to hold classes only on-line via Zoom in the spring semester. Many of them postponed reopening for the fall semester, and some remained closed into the winter due to fears of Covid 19. Some colleges and universities that opened for the fall semester were soon forced to close by the number of students who tested positive for Covid 19. Some of the surrounding communities complained that infected students had spread Covid 19 to the public at large. Schools that were closed through the spring and then the fall found that on-line teaching failed many of their students, especially slower learners and ones whose families lacked electronic means of connecting to their classes. Large proportions of parents began to clammer for the reopening of in-person schooling, while others worried that children exposed at school could bring infections home to vulnerable parents, siblings, and other relatives.

In the second quarter of the year, the nation’s overall economic output dropped at a rate that would amount to about one-third if extended to the full year. While some jobs were started again in the late second quarter and third quarter, some 12–14 million people remained out-of-work in early 2021. By then, new unemployment claims filed each week were again at rates four or five times the rates in the previous year. Some 700,000 people had, at least over the short term, withdrawn from the labor force, especially women supervising young children at home. Year end data for 2020 showed that the GNP had dropped from 2019 and would likely not regain its previous level until late in 2021.

Self-isolation, social distancing, and masking were adopted by many elements of the population, while other elements resisted these measures. In places, conflict, even violence, was reported when people wearing masks were attacked by people politically opposed to mask-wearing. In some parts of the country quarantines were observed by people who displayed no symptoms but may have been exposed to SARS COV-2, while in other parts of the country self-isolation was resisted as a restriction on personal freedom. Newspapers across the country reported increasing numbers of cases of depression, domestic abuse, suicide, overuse of alcohol, and illicit drug abuse, especially of heroin and fentanyl, leading to increased deaths from overdoses. Major cities saw increased rates of homicide due to gun violence, with Chicago and Philadelphia drawing special attention in the press.

In the second quarter, the federal government passed a plan of expenditures to provide essential financial support to the individuals and families who had lost jobs. Support was also extended to a wide range of small enterprises to encourage them to maintain jobs and payrolls. The $2.2 trillion expenditure staved off tragic situations for millions of families suffering unemployment, but Congress and President Trump’s administration failed to follow up after the funds mostly lapsed at the end of July. Democrats and Republicans could not agree on the scale of future measures. The president offered no effective leadership in resolving the disagreements and refused to meet with the Democratic leaders in Congress, in particular the Speaker of the House of Representatives, reportedly because of her role in his earlier impeachment.

The federal government offered surprisingly little effective response to the pandemic, with testing and tracing delegated to states and counties even as numbers of infections and deaths soared above rates seen in other nations with advanced economies. Policy was complicated in its public effects by President Trump’s repeated lying on the actual situation. It became increasingly clear that he was failing to take the advice of public health and infectious disease experts while also obstructing their efforts to report frankly on the scientific understanding of the epidemic. Out of belief that it would reduce the economic harms of the pandemic, the president discouraged masking and social distancing, even holding large campaign rallies with enthusiastic followers sitting and standing, cheering and shouting, mostly unmasked and close to one another. Only near the end of the year did Congress pass another relief bill, some $900 billion, an amount that most economists regarded as much too small. The bill was then poorly implemented. Although it offered relief to some families, its main effect may have been to obstruct the incoming Biden administration’s efforts to obtain Congressional support for more adequate funding.

Seeking to rebuild the economy before the November election, President Trump encouraged states and cities to “open up”—that is, remove restrictions on economic activities. Many governors and mayors in predominantly Republican states responded to his entreaties. While modest short-term economic benefit resulted, Covid 19 then spread to those states, resulting in a new wave of the epidemic and heavy loss of life. It became apparent that African American, Latino American, and Native American citizens were being affected more heavily than other groups, with higher rates of infection, hospitalization, and death. In this respect, the pandemic was linked to the crisis of racial injustice. Analyses indicated that “essential” but low-paying jobs held by disadvantaged groups exposed them to greater risk of infection, but also that they received less adequate health care, indicating an injustice in the nation’s system of health care. When the Biden administration came to power, it was facing a map of the pandemic that showed infections and deaths heavily impacting all 48 contiguous states. A vaccination program had been started, but was poorly coordinated, resulting in far fewer vaccinations delivered into arms than the initial goal.

The three sets of events—the police killings and exposure of racial injustice, the rapid spread of a deadly pandemic, and an acute economic recession resulting from the pandemic – interacted with one another. Together, they engendered a growing sense of national crisis. As the national election campaign progressed, the sense of crisis extended into politics. President Trump repeatedly said that he could not lose to a candidate as weak as Joseph Biden and that, if he did lose, it would only be due to fraud and, hence, he would not concede. After the election, the sense of political crisis deepened further as Trump refused to accept his loss, proclaimed that he had won in a landslide, and never accepted the legitimacy of Joseph Biden’s victory.

Ultimately, his refusal led to the mob invasion of the Capitol, Trump’s second impeachment, and a trial in the Senate in which 43 Republican senators blocked conviction. The failure of the conviction reinforced a crisis regarding Constitutional limitations on uses of federal authority and the respects in which officials are bound in their conduct by their oaths of office to uphold the Constitution, perhaps as deep a political crisis as the nation had experienced since the Civil War.

In the following sections, I use Parsons’ four-function analysis of society into primary subsystems to explore causes of this complex sets of national crises. For each subsystem, I will begin by reviewing some of the longer-term changes that set the context for the more focused crises of 2020, then discuss more immediate developments. I will proceed by analyzing problems in each of the four subsystems, culminating with core issues in the societal community.

## The Pattern-Maintenance System

The pattern maintenance system consists of institutions that generate commitments to basic values of social life, to the broader culture within which the values take on meaning, and to the society as a whole. Each of the other subsystems of society has its own pattern maintenance sector that generates commitments to social action within its specialized institutions. In this context, our concern is with the values that connect individuals and groups to social life in general. A major focus is on the institutions that contribute to “socialization” in the broadest sense: families, schools, colleges and universities, churches and other religious organizations, cultural associations of various kinds – music groups, museums, artistic and literary groups, scientific associations, etc.

Parsons argued that, given the diversity of American society, there is considerable pluralism in the explicit perspectives and doctrines among the socializing entities in American society, yet they generally share a unifying *pattern* he called instrumental activism, a term related to, but more comprehensive than, Weber’s inner-worldly asceticism. The emphasis on instrumental activism continues, I believe, to correctly characterize the society-wide pattern of values. We see members of the society socialized at all levels—in families, schools, churches, universities, etc.—to be activistic as individuals and as participants in various collectivities. Americans seek to improve instrumentalities for social action of all types, including the personal skills used in various social roles and the operations of social institutions with which they are engaged. However, the very activism of citizen’s value-commitments can engender conflict when various groups, associations, or networks hold different concrete values and are committed to advancing their own values, even if against the values of others.

In the present situation, value-commitments have aligned into two opposing complexes of general orientation, as is particularly apparent among religious denominations. A “mainstream” set of institutions, centering on the more liberal Protestant denominations as well as liberal Catholics and Jews, generally accepts diversity regarding race, ethnicity, sexual orientation, career paths, and social outlooks. It generally looks forward to a more open and flexibly structured society, one in which justice and civil liberties in their many facets are extended. It has been more “internationalist”, valuing the contributions of other nations to global welfare and supporting immigration, travel abroad, trade with other countries, and cultural exchanges with peoples around the world. It typically looks to the rationalist ideologies deriving from the Enlightenment and the various sciences as well as professional journalism for information on social and natural conditions. In recent decades, it has favored protection of the environment and efforts to delimit the damages from climate change – in 2020, storms in the South and massive wildfires in the West. It has generally adopted a stance of rationalism and rational criticism in its perspective on secular institutions and social policy.

A more “fundamentalist” orientation centers in evangelical movements within Protestantism, especially the Southern Baptist Church, with conservative Catholics and some Orthodox Jews adhering to similar outlooks on secular affairs and institutions. Many adherents of this broad orientation have college educations, but a majority does not. Many with college degrees earned them at institutions affiliated with fundamentalist Protestant denominations or the Catholic Church. Even those with college degrees often place less emphasis on education, especially in secular fields. Distrust in the sciences and in scientific findings, especially in the biological sciences with their grounding in Darwinian natural selection, is common. Belief in the literal truth of the Bible, including accounts of “miracles” is not uncommon, as is belief in the “Devil” and evil associated with him. Distrust in secular moral standards and institutions, including government, is not infrequently voiced, especially when thought to be antithetical to Biblical teachings. Trust in social traditions complemented by distrust in rationally developed or science-guided proposals to improve basic social institutions is common.

Some members of the fundamentalist alignment articulate, in various degrees, ideas of white supremacy and white nationalism. Many are distrustful of the increasing numbers and rising social status of people of other racial and ethnic groups. The prediction that the U.S. will have a non-white majority in its population within a few decades or less has often provoked their ire. They tend to express suspicion toward immigrants from cultures outside of Europe. Strong patriotic commitments are sometimes tied to an ideal of returning to earlier, putatively more peaceful, intergroup relations, commitments that often shade into explicit valuation of economic and political dominance by whites. Many vouch for the virtues of blue-collar jobs, working class lifestyles, and the neighborly relationships of small towns and working class sections of cities. There has been long-standing opposition to abortion and, for some, even birth control. Openly gay individuals, gay marriages, gay lifestyles, and transsexual individuals are often viewed as incompatible with tradition. In recent decades, the distrust of science has come to a focus on opposition to the science of climate change and its consequences, including fear of lost jobs in the coal, oil, gas, and chemical industries.

The differences between the two broad sets of orientations toward secular life and institutions have fluctuated for decades between covered over and sorely open. At times, they have receded in importance as various leading figures have emphasized common values and commitments, as did Jimmy Carter and to a degree Bill Clinton during their presidencies. They were able to advocate for ideals of patriotism, Americanism, and democracy, including respect for wide ranges of opinions, without precipitating sharp dissension among either mainstream or fundamentalist believers. President Obama emphasized mainstream rationality in a manner that resulted in sharp opposition among fundamentalists. President Trump’s leadership has activated the divide more acutely. He has emphasized his membership in the “elite” as an Ivy League graduate, a billionaire businessman, and mainline Protestant, but he has also attacked the “elite” and its orientation as threatening the welfare of common people. He has sought the support of fundamentalist Protestant ministers. He has avoided rejecting and at times encouraged the support for white supremacist groups. He has repeatedly hinted at agreement with their views while yet trying to cover forthright endorsements in coded terminology. He has explicitly opposed the Black Lives Matter movement, alleging that it is allied with violent adherents of “Antifa”, which he has claimed to be much better organized and more widespread than law enforcement reports. He has refused to acknowledge climate change and has dismissed scientific evidence of it without offering clear reasons or evidence.

In sum, a major difference between the two patterns of religious orientation concerns the status of secular culture and institutions. The mainline orientation accepts secular practices and institutions as legitimate insofar as they are rationally grounded. It remains open to policies that promise to institute social change improving various aspects of race relations. The fundamentalist orientation, by contrast, tends to view secular thought and policies with distrust except when linked to traditional religious principles and institutions. In the present era, with its range of substantial social changes, especially regarding racial, ethnic, and religious diversity, the fundamentalist orientation engenders suspicion. Sectors of it have given rise to explicitly white supremacist and nationalist movements opposing the legitimacy of especially Islamic, but also Buddhist, and Hindu religious practices as well as non-white racial groups. In some cases, white supremacist groups have propagated conspiracy ideologies alleging that officials of the government, especially Democratic leaders, have been promoting nefarious policies designed to undercut the advantages and privileges of white citizens. Q-Anon has been perhaps the most prominent such conspiracy ideology. Trump refused to reject the support of its adherents during the campaign; at least two of its adherents were elected to Congress in 2020. Various so-called “militias”, typically with links to conspiracy theories, have arisen in opposition to actual or proposed federal policies, such as, gun control measures, restrictions on use of public lands by private parties, or less restrictive laws on abortion. Members of the militias often participate in public demonstrations while armed with military-style weapons and have at some events threatened violence in support of their racial and cultural outlooks. The January 6^th^ invasion of the Capitol seems to have involved a complex mix of fundamentalist, white supremacist, conspiracy theorist, and militia groups, some appealing to “1776” as a symbol of a revolution they hoped to instigate.

African American denominations, such as the African Methodist Episcopal Church and the American Baptist Church, have been closer to the fundamentalist orientation in terms of theology and modes of worship, but mainstream and liberal regarding social policy. They are proud adherents of the Civil Rights Movement and the mission of Martin Luther King, Jr., hence supporters of advancing racial equality in all spheres of life. However, some followers hold that the religious leaders of these denominations had become too comfortable over past progress and too accommodating to predominant social conditions until the reactivation of the Black Lives Matter movement over the summer. In the current crisis, African American ministers have become key spokespeople for more comprehensive racial justice.

Educational institutions, in the ways in which they prepare individuals for adult roles in American society, are also a source of division. There is a substantial range of difference in the quality of public schools, including charter schools. Citizens raised in poor urban areas, especially in predominantly African American neighborhoods, and in rural school districts tend to be less well prepared for higher education than citizens raised in middle class, especially upper middle-class, urban neighborhoods and prosperous suburban communities. Working class and poor urban school districts and rural school districts tend to be stressed in terms of resources and quality of teachers. Notable percentages of students from poor families fail to complete high school—or do so only later in their adult years through GED programs—and are disadvantaged on the labor market. Among those who complete school in poorer systems, rates of college attendance are low and the colleges they attend are typically ones with curricula focused on preparation for specific vocations—teaching, nursing, bookkeeping clerical work, etc.—rather than a broad general education and the sciences. Vocational education, whether in high school or community college, provides less preparation for citizenship engaged with issues of public policy. Although the American educational system has advanced greatly over the decades since WWII in its ability to provide high quality education to larger proportions of age cohorts, and especially in the preparation of scientists and technologists, gaps in quality remain prominent, both in public schools and in colleges and universities. Where colleges are affiliated with evangelical or fundamentalist churches, they not infrequently reinforce fundamentalist religious commitments, including skepticism or disbelief regarding science that may seem to contradict Judeo-Christian ideas of Creation.

American families have traditionally institutionalized important sex-role differences between marital partners. Men have been regarded as the primary “bread-winners” and family decisions, such as, regarding location of residence, tend to be made to advance their careers. Although women are now much more frequently employed than in previous generations, they tend to adapt their work roles to ones that are available to them, given family commitments to their husbands’ jobs. Women are generally more heavily involved in household tasks and in child-rearing than are their husbands. These family patterns have more recently come under challenge in the era of the Women’s Movement. Increasing numbers of families are placing greater emphasis on the wife’s employment and career. However, this new pattern is often adopted after family conflict, and it remains rare among families with fundamentalist backgrounds.

Pattern maintenance functioning thus involves a broad divide between mainline or liberal and fundamentalist or conservative patterns. The divide appears to have long-lasting religious foundations but extends importantly into the educational and familial domains as well. The two patterns thus characterize two general modes of orientation to social life as a whole and two broad outlooks on American society and social institutions. As we will see, the division in orientations extends into the other subsystems of society. During the period of Trump’s presidency, the division was mobilized into the political domain in ways that precipitated open conflict where it had previously been more latent.

## The Economy

Long-term changes in the economy have affected the livelihoods and lifestyles of large proportions of the population. Some sixty and seventy years ago, factories were moved from the “rust belt” cities and towns of the Northeast and Midwest to the South and Southwest, leaving large areas with fewer jobs and lower paying work. Major industries, such as steel production, car manufacturing, spinning, weaving, and clothing factories, coal mining, petroleum refining, and chemical plants were affected, leaving many workers without the employment for which they had proudly developed and used personal skills. In recent decades, globalization has moved jobs of similar kinds to other countries, including work in the newer high-technology industries. In these transitions, a principal factor has been that work migrates to where the inputs of labor, if appropriately skilled, are cheaper. Corporations have located their operations where they can be conducted most cheaply, shifting many types of work from the U.S. to other nations. Japan, Germany, and, more recently, China, India, Indonesia, Mexico, Taiwan, and Vietnam, have developed the capacities to compete in industrial production and in high-tech industries, such as, manufacture of electronic chips, assembly of computers and cell phones, and operation of large-scale computer networks. The result has been widespread impoverishment in the “rust belt” inner-city communities and even in the communities in the South and West where jobs were first moved decades ago. Deteriorating housing, greater welfare dependence, reduced public services, the insecurities of rising crime rates, discouragement about future careers, opportunities, and personal well-being, and more frequent drug and alcohol use have resulted. The consequences have been felt in small towns that have lost their factories as well as in large cities. They have also affected farming, especially family farming, as consolidation into more efficient large-scale operations have made small farms economically non-competitive.

Over the same decades, new technologies and their efficiencies have also affected levels of employment. An example is the robots that perform the welding on automobile frames and bodies, which have removed previously skilled jobs from assembly lines. Computer-guided machinery now accomplishes the same work more quickly, precisely, and cheaply. Similarly, offices equipped with networks of personal computers have supplanted many clerical and secretarial jobs. Automated processes of managing stocks of goods at retail stores have, again, supplanted jobs. Companies, such as Amazon, that ship vast varieties of retail goods directly from warehouses to customers, thereby supplanting retail shops, have had negative, and growing, impacts on levels of employment, even though they have been hiring hundreds of thousands of workers.

The globalization of industry and the technological changes are importantly interrelated. The development of huge container ships has greatly reduced costs of shipping goods to American markets from Asian suppliers. The nearly instantaneous, cost-free communication made possible by the Internet facilitates exchanges of information that contribute to the globalization of production processes and supply chains.

At the same time as much of the labor force has been squeezed in terms of opportunities and income, the financial sector has risen to a new level of dominance in the economy. Its increasing prominence has precipitated rising distrust of “Wall Street” and sympathy for declining “Main Street” small businesses. With the capital from large banks, investment banks, and hedge funds, “Big Box” retailers—supermarkets, pharmacies, and hardware stores—have forced small businesses to close when they cannot compete in terms of prices and varieties of goods. Many small towns and cities have seen their old commercial streets radically changed as store after store closed, leaving rows of abandoned or underused storefronts lining once active centers of local commerce. Shopping has relocated to impersonal “big box” stores on cheaper land in the outskirts. Although families can purchase more and sometimes better merchandise at the new stores, the loss of locally owned shops is experienced as harming civic life.

Small farms throughout the country have also faced challenges, in part due to the growth of “industrial” farms that produce meat, milk, vegetables, and fruits on a scale and with an efficiency that small producers cannot match.^1^ The cost of tractors and other farm equipment, as well as other necessary capital investments, for example, modernizing barns and purchasing additional land, have made it difficult for the small-scale producers to compete with large operations. Price fluctuations for farm products have also made it more difficult for small farmers to anticipate income and ride out periods of low prices. A result has been that in much of the country many farmers have closed down, selling their land and equipment, and finding other work to support their families.

Millions of jobs have been lost across the country through the processes just described. They have left large proportions of the working class and lower middle class feeling that they have lost the economic security and well-being that they had earned and are their due. Many people are working at jobs in retail, fast-food, or other low-wage jobs, often making half the wages they had earned in factory jobs or even less. Many lack the savings to afford rent or mortgage costs if they are out of work for a period of time or if there are unanticipated costs for an illness, a car repair, home repair, or replacement of a household appliance. They are thus searching for ways of getting back on an economic track that will cover costs of family necessities and perhaps make it feasible to leave a small apartment and purchase a home.

While so many families face economic struggles, it is apparent that other sectors of the economy are experiencing extraordinary success. The consolidation of large corporations has resulted in chief executive salaries that are often as high as $10 million or $12 million, plus year-end bonuses of millions of dollars of stock. Banking and finance have been expanding and consolidating their control over other sectors of the economy. Anyone who reads the newspapers knows that there are huge numbers of people gaining great wealth through hedge funds, private equity, the major investment banks, and the large classical banks. Private equity is often used to buy out firms with considerable capital value, burden them with the debt needed to purchase them, then cut costs by laying off personnel and selling off real estate and other capital assets. A not uncommon result is to leave the resulting companies at risk of bankruptcy during economic downturns. The consolidation of firms almost invariably results in the firing of employees in the name of efficiency, typically by laying off specialists in accounting, purchasing, human resources, etc. who become “redundant” when parallel offices are consolidated. Even when the financial firms fail, as happened in the Great Recession through faulty “packaging” of improperly valued mortgage securities, the principals seem to come through the crisis with retained wealth. For people who lost work in the recession or lost their homes due to mortgages they could not pay and, in the economic downturn, could not sell at prices covering their mortgages, the continued prosperity in the financial sector was galling. When the newspapers report on prominent individuals said to be worth hundreds of millions or billions of dollars, sometimes with expensive homes in a few American cities and perhaps in London and Paris too, the resentment of people who have lost their livelihoods, homes, and savings is understandable. Many citizens are aware that lawyers in the large firms serving the financial industry have also become very prosperous. Fees of $1,000 per hour for senior partners, perhaps $350 per hour for young associates, are common.

The “tech” industry has also expanded conspicuously and become a major generator of wealth. The tens of billions of dollars gained by the principal executives of Microsoft, Apple, Google, Facebook, etc. are well known to the public. Beneath these executives are software and hardware design engineers whose compensation and bonuses, over years, amount to millions of dollars. Early employee/stock-holders of these firms or early outside investors are often very wealthy. That a large proportion of citizens, as well as many millions of people in other countries, routinely interact with these corporations makes the wealth they generate highly conspicuous.

In sum, the American economy has proved dynamic over the last several decades. Processes such as implementing new technology, relocating processes of production, refinancing operations to expand them, consolidating formerly separate corporations have in most cases increased efficiency. Consumers have benefitted from new, better, and cheaper products, even if they are now increasingly produced in other countries. Yet, much of the economic efficiency has entailed loss of jobs, and when the affected workers find new work, they are often in lower-paying jobs. The intense competition among workers to obtain jobs has led to strong downward pressure on working class wages. A substantial proportion of the work force finds itself underpaid and living under conditions of financial stress. At the same time, many who feel financial stress are quite aware that other strata have been achieving extraordinary economic success and wealth. The resulting economic inequality may be the greatest since the Robber Baron era of the late 19th and early 20th centuries.

These long-standing processes have been exacerbated in 2020 by the economic effects of the Coronavirus epidemic. Sectors of the economy, especially small businesses, such as, bars and restaurants, personal services, such as hair-dressing, and various shops, have in many states been closed, or largely closed, for much of the year. In April and May, some 23 million people became unemployed and some 700,000 individuals withdrew from the labor market. Federal support sustained many small businesses through July with the result that many jobs were maintained through the early months of the crisis. Over the summer and early fall, nearly half of the unemployed regained jobs, although often ones less well paid than their previous jobs. A sense of injustice was heightened as people in finance, “tech”, and some other fields continued to thrive, while others faced continued unemployment or underemployment, difficulty in paying bills, repossession of cars, dependence on foodbanks for nutrition, and threatened loss of apartments or homes.

As these economic changes proceeded, African Americans and Latinx were often exposed to the sharpest declines in income. The decades since the Civil Rights Movement saw an increase in racial minorities employed in middle class positions, yet large proportions remained in just the kinds of jobs exposed to declines in real wages. Day laborers, landscape workers, non-union factory workers, check-out clerks, shelf-stocking assistants, restaurant servers, and so forth all experienced declines in inflation-adjusted wages over recent decades. During the pandemic, they have often been among the first to be furloughed or laid off, frequently with little idea about whether or when they might be recalled. Their economic situation likely fostered an openness to mobilization into the Black Lives Matter demonstrations, although in most communities the demonstrations were led by middle-class women and men. As the Covid 19 emerged and then spread more widely across the country, the same types of jobs, some of them classified as “essential”, proved to involve more frequent exposure to infection. Rates of infection proved in many parts of the country to be notably higher for African Americans and Latinos than for middle-class whites.

## The Polity

The polity is the system of legally regulated formal organization that implements the collective goals of the society and its various segments, including states, counties, and municipalities. It includes the political processes that determine collective goals as well as the processes that realize the goals through executive and administrative functions. Leadership is a basic element of the polity, as high officials at every level of the federal system hold their positions by virtue of public advocacy for specific goals and means of attaining them. It is the public advocacy of leaders that mobilizes political support among the citizenry and thus the collective impetus to pursue specific policies. Yet, political systems, and especially its leadership elements, are, like other systems of social action, fallible. There may be errors in identifying goals and in implementation processes; policy implementation may fail or produce unanticipated consequences, thus harming some or all elements of the citizenry. Political processes are for these reasons inherently controversial, and in large democratic societies the controversies may engage deep social divisions, as it has increasingly been in the U.S. over the last several decades.

Under the Constitution, the American political system has a federal structure with authorities divided among the national or federal government, the 50 state governments, and a myriad of local governments. In aggregate, state and local governments employ far more officials and provide a wide range of services, although just which services varies across the country. Yet, the federal government, its leadership, and its policies set a general tone for politics throughout the nation while also providing many essential services.

Historically, the expansion of federal operations and services during the period of predominantly Democratic leadership from the Great Depression through World War II and the Cold War established the policy framework of modern government. Important changes included the establishment of welfare policies, Social Security, Medicare and Medicaid, creating agencies to provide more active regulation of finance, industry, and commerce, including working conditions in factories and offices, expansion of the State Department and diplomatic activity, and growth in the size and scope of the military and its capabilities. The establishment of new levels of opportunity for African Americans through migration from the South to the North and West, through the nationwide activism of the Civil Rights Movement, and through the important legislation of the mid-1960s also brought about important social change.

American politics also underwent a reaction to the Civil Rights legislation. President Nixon gained election through a “Southern strategy”, emphasizing States Rights and the shifting of some powers to state and local governments as grounds of resistance to federal laws and policies advancing racial justice and equality. His movement reshaped American politics for the next several decades by switching the allegiance of conservatives in the South from the Democratic to the Republican Party. Nixon also favored restrictions on federal spending and the scope of new federal policies, although he did initiate important environmental policies.

The changes started under Nixon were extended after President Reagan took office in 1980. Reagan emphasized greater restrictions on the federal budget, reduction in tax rates, especially for the wealthy, limiting the powers of the federal government, and removing many regulations affecting the economy to favor a “free market”. Reagan summarized his ideological perspective with statements to the effect that government is not the solution, but the problem. Proclaimed a political saint by conservative Republicans, the ideology Reagan advanced has predominated in American politics to the present.

To sustain their political competitiveness despite a demographic base declining as a proportion of the electorate, Republicans have pursued policies that have narrowed the democratic cast of national politics. Where they have controlled state legislatures, they have gerrymandered both legislative and Congressional districts to dilute the effects of Democratic majorities in urban areas. They have initiated identity-card requirements for voters in efforts to keep Democratic-leaning groups from voting. Exploiting the equal representation of Republican-leaning states with small populations in the Senate and combining it with the filibuster, they have blocked popular Democratic legislation, saddling Democrats with records of ineffective governance.

Democratic presidents since 1980 have promoted new policies to address social problems, but, facing strong Republican opposition, often disproportionate to its actual political support, they have had to rein in their ambitions. President Clinton gained election by campaigning as a moderate and, in office, strengthening the moderate wing of the Democratic Party. President Obama’s response to the Great Recession was limited by his efforts to gain Republican support through compromise. Republican resistance, emphasizing limits on federal budget deficits and hence expenditures, slowed the recovery from the recession with the political consequence that Democrats lost control of Congress. Obama’s achievement of a new health insurance policy—a matter on which the Clinton administration had failed—was only won in the face of stiff Republican opposition, including a summer-long pretense to offer support in exchange for reductions in the scope of the legislation. The divisions between the parties deepened when the Republican Senate Majority Leader, Mitch McConnell, refused to consider President Obama’s nomination of Merrick Garland for the Supreme Court.

President Trump campaigned for office during this period of political division. His campaign strategy was further to exploit the divisions. At one level, he breached long-standing political proprieties. He had gained public attention as a political figure with assertions that President Obama had been born in Africa, and not being native-born, was not qualified to be president—improbable claims he did not renounce even after they were disproved by publication of Obama’s birth certificate. Trump amplified his strategy of delegitimating opponents by repeatedly defaming his opponent, Hillary Clinton, by encouraging shouts of “Lock her up” at his rallies. He attacked the Obama administration and citizens supporting it, especially racial minorities, in terms that had long been regarded “beyond the pale”. At a deeper level, Trump sought, with his “Make America Great Again” slogan, to mobilize the support of citizens who felt alienated after losing prosperity and status through the economic and social changes of the previous decades. These groups included small farmers, blue collar workers in industry, residents of rural communities, small business owners, adherents of the evangelical movement, and “white supremacists” worried by the increasing proportions of racial minorities in the citizenry. Perceiving that Trump was leading his supporters to overturn basic democratic institutions, Hillary Clinton called them “deplorables”, a conspicuous error as it confirmed to many that she condescended to, and gave little consideration to, their outlooks and interests. Her statement consolidated the democratic legitimacy of Trump’s appeal to followers disaffected from the the Obama administration and the Democratic Party.

After his election, Trump continued to focus on his coverage by the mass media, including Twitter, Facebook, and YouTube as well as Fox News and select newspapers, more than on government policy. Perhaps more than any other president, he made his own messages as well as commentary about them, by supporters and by opponents, part of the citizens’ daily engagement with the media. Citizens were confronted with the presence of the president more unrelentingly than had ever been part of the country’s civic life. Trump sought to sustain the MAGA campaign message with its appeal to citizens who believed that the America of decades past was morally superior to its present condition. He proclaimed an “America First” foreign policy. Some of his hastily implemented measures proved to be legally invalid and had to be revised before they could be implemented, notably his effort to ban immigration, even travel, from many Muslim nations. To address the removal of factories and jobs to Mexico, he renegotiated NAFTA and renamed it to emphasize that the new terms were more favorable to the U.S., even though the changes were smaller than he claimed. He started a “trade war” with China, imposing a range of tariffs to reduce imports from that nation. He falsely claiming on many occasions that Chinese exporters paid the tariffs, not American importers. He pursued the project of building a wall on the Mexican border, originally announced during his campaign, despite resistance from Congress. After reallocating construction project funds from the Defense Department budget, work on the wall began. As a theme of his 2020 campaign, Trump exaggerated how much of the wall – actually, a tall, but easily breached fence—had been built.

A key element of the America First policy was to remove the U.S. from international agreements: the Paris Accord on limiting climate change; a trade agreement with Pacific nations, which allowed China to advance an alternative regional agreement; nuclear and ballistic missile arms agreements with Russia; and the agreement limiting Iran’s development of nuclear arms. Trump criticized the European Union, sought to reduce trade with Union nations, and encouraged Great Britain’s withdrawal from it. He also criticized NATO and, on several occasions, insulted the political leaders of NATO allies. The America First measures were intended to appeal to citizens who believed their interests had been harmed by globalization and accommodations to it by previous administrations. However, on several occasions, he deferred to Russian President Putin on matters regarding which American intelligence officials had provided contrary information. He also claimed to have developed understandings with Kim Jong-Un about limits on North Korean nuclear weapons, even though Kim declined to affirm and later rejected the claims. These events called into question Trump’s abilities as a negotiator in international agreements and the truthfulness of his statements about them. The tensions Trump engendered in relations with European allies led them, implicitly if not explicitly, to reject American leadership, regarding it as no longer trustworthy.

In the domestic realm, Trump pursued long-established Republican policies: tax cuts favoring corporations and the very wealthy while highlighting quite modest ones for the middle class; nominating conservative candidates for judgeships on federal courts, including the Supreme Court; restricting immigration and increasing deportations of undocumented immigrants; removing federal economic regulations that were claimed to hinder economic growth; promoting the development of the petroleum and gas industries; undermining the Obama administration’s policies to limit climate change; opposing abortion; and extending Second Amendment-related rights to gun ownership and use. Overall, it was not a robust set of policies for a presidential administration, but President Trump advocated them persistently on television interviews, through Twitter messages, and on Facebook and YouTube.

Beneath the “America First” and “Make America Great Again” were implicit appeals of substantial importance. The theme of returning to a previous era of American greatness carried racial and ethnic overtones. Many white citizens, especially those who had in recent decades been experiencing relative economic decline, understood Trump to be resisting the increasing role of African American, Latino American, and Asian American citizens in the nation’s cultural, social, and political life. Over time, explicit white supremacy became more prominent among Trump’s adherents. Trump’s advocacy of gun rights gained response especially in the South and West, resulting during his administration in the growing size, activity, and prominence of various “militias”. The militias, especially the Proud Boys and the Oath Keepers, were to become important in responding on Trump’s behalf to the Black Lives Matter demonstrations in the summer of 2020, in participating in rallies during his campaign, and in his final effort to overturn the election results on January 6, 2021. During 2019 and 2020, Trump began to court the support of conspiracy theorists, many of them tied to militias, with the QAnon movement being the most prominent. A common theme of the conspiracies was that Democratic leaders, such as, Hillary Clinton, Nancy Pelosi, and figures in the Obama administration, were tools of the Devil, promoting evil. A unifying element of MAGA was an appeal to evangelicals, including Biblical literalists who believed in the Devil. Although Trump did not present himself as an especially religious person, he appealed directly to prominent pastors of the evangelical movement, accepted their broadly framed social and political support, and seemed to relish in it. Only in the last months of his administration did any leading evangelical pastors speak out against Trump’s racial appeals, and they were few. Approximately 25% of the electorate was drawn into fervent and constant support of Trump through the 2020 election on the overlapping bases of evangelical commitments, anti-abortion appeals, conspiracy theories, gun rights ideology, racism, and even anti-Semitism.

Trump overstepped his authority or compromised his leadership on a several occasions. The first instance was his statement that there were ‘good people’ on both sides of the right-wing Charlottesville rally, during which, in the face of liberal opposition, some had shouted “Jews will not replace us!” along with racist chants. A second was the separation of children from immigrant parents at the Southern border and then housing the children in atrocious conditions. A third was the phone call with the Ukrainian head of government in which Trump threatened to hold up American military aid until Ukraine announced an investigation of alleged corruption by Joseph Biden and his son Hunter. The threat was followed by an actual hold up of aid until after the events had been reported in the press. Trump was then impeached over the events, but the Senate failed to convict him. A continuing matter involved investigations into the Trump campaign’s connections with the Russian government’s efforts to prevent Hillary Clinton’s election as president and thus its effort to assist Trump’s 2016 campaign. The investigations were started under the Obama administration, sustained by the FBI, and then transformed into the Mueller investigation. As Mueller’s report made clear, Trump obstructed the investigation at several points and could have been held legally responsible had he not been in office. Several of Trump’s political allies and campaign officials were later prosecuted for their involvement with the Russian election interference and/or their obstruction of the Mueller investigation. Attorney General Barr’s mischaracterization of the Mueller report saved Trump from greater political embarrassment and perhaps from an impeachment. However, Trump’s characterization of the relations between his campaign and Russian agencies and then the Mueller investigation became a continuing focus of his many lies. After the political controversies over the investigation, Trump continued to lie. By the end of his administration, he was lying on nearly all matters of public controversy. The Washington Post counted more than 30,000 misstatements and lies over the course of his time in office, an historically unparalleled corrosion of presidential communications with the public.

The killing of George Floyd by police officers during what might, without violence, have been a routine arrest set off a national wave of Black Lives Matter demonstrations. Millions of citizens, whites as well as Blacks, marched peacefully, often many times, to show their support for African American citizens. In some cities, looters exploited the attention that police gave to the demonstrations to attack stores, cash machines, and some public buildings, creating fears of a loss of public order. When additional cases of police killings of African Americans came to light, demonstrations were renewed and, in a few cities, they continued through the summer and fall. The overall impact of the renewal of the Black Lives Matter movement was substantial. Most Americans began to recognize more clearly that the society still sustained racial injustices with a history that dated back to the nation’s very founding. Many institutions made changes to create new opportunities for African Americans and other people of color. However, the federal government seemed unresponsive to the new awareness of injustice. The president and attorney general mounted repressive measures complemented by exaggerated and unrealistic allegations about violent left-wing leadership of the demonstrations. The president used federal officials forcefully to end a large and peaceful Black Lives Matter demonstration near the White House so he could walk to a nearby church and be photographed holding up a Bible. These events made apparent the administration’s lack of concern about issues of justice and of full and fair citizenship for African Americans and other racial minorities.

It was in this context that SARS COV-2 expanded into a pandemic affecting the entire nation. Initially, President Trump dismissed its importance, claiming that there were only a small number of cases in the U.S. and all would recover within days. Later, he acknowledged that Covid 19 was more widespread, yet maintained that it was no more serious than ordinary ‘flu. In a next phase, he declared “war” on what he recognized would become a serious epidemic, while also suggesting it was a problem of the Democratic Northeast, especially New York City. When governors and mayors, following the advice of epidemiologists and infectious disease specialists, began to close schools, stores, restaurants, and other services, he called the pandemic another Democratic “hoax”. After the error of seeming to advocate, on television, ingestion of disinfectant as a cure for Covid 19, he turned leadership of the federal response to Vice President Pence and withdrew from attending to the pandemic and related policy issues. As his presidential campaign became more active, he began to emphasize that responses to Covid 19—closing schools, shops, restaurants, and offices social distancing, and masking—would harm the economy and thus undermine his major campaign theme, namely, that he had promoted the most successful economy ever. At times, Trump even attacked government infectious disease and public health specialists and opposed their scientific reports. The Department of Health and Human Services, the Centers for Disease Control, and the Federal Drug Agency, under criticism and limitation by the White House, proved remarkably ineffective despite their vaunted scientific capabilities. During the campaign and after, Trump largely ignored the hundreds of thousands of deaths and millions of cases of disease due to the pandemic. Many political commentators argued that the weak response to the pandemic was the chief factor in Trump’s loss in the election.

The effects of the Covid 19 pandemic had a variety of political effects across the country. The negative response of President Trump and many of his followers to such public health measures as masking, social distancing, and closing bars, restaurants, and shops precipitated political controversies in many communities. Many states and cities found themselves in budget crises due to losses in tax revenues. Across the country more than one million state and municipal employees were furloughed and various public services reduced. In parts of the country, state and local leadership was ineffective, partly as a result of aligning policy with themes in the president’s tweets and speeches. Where governors and mayors offered effective leadership, they were frequently criticized by the president. The incompetence of the federal administration became increasingly apparent. The nation fell into something like a political depression, where the capacity of political institutions to manage public affairs and achieve collective goals effectively was substantially diminished.

On the left, policies have increasingly been pitched to the interests and culture of the Atlantic and Pacific coast states and to the major metropolitan areas while tending to overlook the interests and concerns of states in the middle of the nation. A consequence has been the alienation of millions of voters who would benefit from many of the proposed policies. On the right, there have been groups that proclaim white nationalism and racism, oppose the so-called cultural elites, and attack the political left as unpatriotic. These differences have deepened during the Trump presidency. His manner of leadership—the telling and tweeting of misinformation and frank lies, the personal attacks on opponents or critics – was corrosive to political and civic discourse. Many of his policies also undermined governmental effectiveness, including the undoing of previous measures to limit climate change, the limitations on immigration, banning of Muslim immigration from several nations, attempting to build a “wall” on the border with Mexico, breaking of international treaties, such as the Paris Agreement and Pacific Trade Agreement, the attacks on NATO members for allegedly not paying sufficient “dues”, changing trade negotiations with China and the European Union, ending participation in the nuclear arms treaty with Iran, and the false claims about nuclear negotiations with North Korea.

A more immediate and intense political crisis emerged in the aftermath of the 2020 presidential election. As votes were counted in the most competitive states – ones that Trump had won in 2016 – it became apparent that he had lost the popular vote by over 7 million votes and the electoral college by 307 to 232, the same margin that he had declared a “landslide” in his victory over Hillary Clinton. Trump refused to accept his defeat and began to declare that he had won by a landslide. He and his supporters filed 61 lawsuits in local, state, and federal courts, and in state and federal appeals courts, including the Supreme Court. Trump’s side lost 60 of the suits, many in which the courts declared that no coherent evidence or legal argument had been presented. The one case in which Trump won, in Pennsylvania, the court noted that the number of votes in contention was far too small to affect the outcome of the state’s vote. Supporters of Trump popularized the slogan “Stop the Steal” and apparently persuaded most Republicans that the election had been stolen and that Biden’s victory and later assumption of office would be illegitimate. The slogan continued to be promoted by Trump followers well into the new year, even after Biden’s inauguration.

As his court losses mounted, Trump intervened directly with state officials and/or state legislators in Pennsylvania, Michigan, Arizona, and Wisconsin in efforts to change the outcomes of the presidential election in those states. News later emerged that Trump had pressured the governor, secretary of state, and other election officials in Georgia to change its outcome in the election. In a recorded phone call, Trump had asked the secretary of state to “find” just the number of votes he would need to win Georgia. Just what Trump may have said to officials in the other contested states remained unclear.

On January 6, Trump and key supporters held a large rally near the White House. Trump, White House officials, and leaders of some supporting groups had invited the Proud Boys, Oath Keepers, and other militias, to attend this culminating rally to oppose the certification of the Electoral College votes by Congress. At the rally, Trump and other speakers, including his older sons, his attorney, Rudolph Giuliani, and some Congressmen, urged his supporters to demonstrate at the Capitol in opposition to certification of the Electoral College vote. Some of the speakers’ language seemed to incite an actual attack, especially when addressed to a crowd that included militia members. Video shows militia members reacting to Trump’s speech by starting to march toward the Capitol and promising an attack. A mob invasion of the Capitol ensued, threatening safety of members of Congress, destroying federal property, injuring police officers, and delaying certification of Biden’s victory by some six hours. The president’s appeal to supporters seemed a last-ditch attempt for him to retain power – in effect, a weak effort at a coup d’etat. The resulting attack at the Capitol precipitated a political crisis unlike any since the Civil War. Impeachment of Trump just days before he would leave office followed. The division between Democrats and Republicans deepened, creating new challenges for the incoming Biden administration’s efforts to implement a sharp change in federal policies and promote national unity.

The trial in the Senate did not resolve the conflicts. The House managers presented a devastating case against former president Trump, but 43 Republican senators voted for acquittal, even though some of them, notably Minority Leader McConnell, stated afterwards that Trump had been responsible for “provoking” the attack on the Capitol and certification of the Electoral College vote. The senators stated that they regarded impeaching a president who had left office as contrary to the Constitution, even though a substantial majority of Constitutional scholars had publicized contrary opinions. The nation was left to consider the Republican Party as still under the sway of a leader whom many in the party believed to have committed a grave crime against the Constitution and American democracy.

In the post-election period, President Trump seemed to give little attention to the crises of racial division, the pandemic, and the economy, which had begun to decline again after a brief period of modest improvement. He played golf, kept himself out of sight in the White House, refused to speak to the public, replaced officials whom he believed to have been disloyal to himself, and planned ways to stay in office, but left the nation without effective leadership. When Biden was inaugurated, the nation had experienced three months of deepening crises.

## The Societal Community

The societal community is the subsystem of society that functions to create and maintain relations of solidarity among units of the society. It is an analytic concept that pertains to aspects of the relations among all of the units that make up the society, individuals, kin groups, local communities, local or national associations, corporations, industries, states and regions, and so forth. It encompasses the aspects of solidarity that Durkheim distinguished as mechanical and organic and Toennies and others in the German tradition distinguished as *Gemeinschaft* and *Gesellschaft*.

Parsons argued that the societal community combines aspects of both mechanical and organic solidarity into one system. At one level, there is a solidarity that derives from a shared sense of the values, general norms, and purposes that engage all members of the society. At another level, there is a solidarity that derives from the interdependence of functionally differentiated or segmented units of the society – the interdependence that derives from say, physicians having available to their families the food produced by farmers, shipped to distributors by truckers, and, eventually, placed on supermarket shelves by store workers and, vice versa, the availability of medical services to each of these other classes of workers. Solidarity is thus generated by relationships of normative similarity or commonality and the interdependence of differences among specialized roles. Some of the specialization involves segmentation, the repetition of similar roles in different parts, especially geographical, of the larger society, as in the case of primary care physicians who perform similar roles, but serve different populations across the country.

One complication not always noted is the that organically solidary *Gesellschaft*, with its interdependence among many differentiated units of the society, includes many thousands of components that themselves are mechanically solidary or small *Gemeinschafts*. Thus, there are strong senses of shared community in villages and towns, cities and suburbs across the country, but also in industries and professions, such as coal mining, iron and steel workers, financial advisors, real estate agents, computer programmers, molecular biologists, and in cultural groupings, such as graduates of particular schools, colleges, and universities, admirers of Country and Western music, and so forth. Each of these small *Gemeinschaft*s provides a basis of fellow feeling, of loyalty, and of cultural togetherness. Each has its own specialized and partial “collective conscience” or set of moral standards and sensibilities as the basis of its solidarity. For the most part, these specialized moral orders are integrated with the moral-ethical grounds of solidarity of the whole society, reflecting aspects of it. The particular groups understand their outlooks to be part of Americanism as a whole, hence expect, despite their distinctive bases of solidarity, to be viewed as legitimate and valued parts of the society. Yet, for some groups, and especially in periods of social stress, just how, and to what degree, they are related to the broader solidarity of the nation may seem problematic.

We may hypothesize that a vast majority of the solidary groupings hold general expectations of receiving recognized status and respect within the broad *Gesellschaft* of American society. Expectations of what level of status and respect obviously vary among entities and their understandings of how they relate to the larger society. Corporate executives in charge of vast organizations with tens or hundreds of thousands of employees, scientists whose discoveries have created major subfields of their disciplines, or leaders of major religious denominations may expect levels and types of recognition that the owner of a small farm or the checkout clerk at a supermarket does not expect. Part of what Americans mean when acknowledging certain social roles, economic statuses, lifestyles, and ways of participating in civil society as “middle class” is that there is a positive valuation, hence respected recognition, of their general belonging. However, where the *basis* of a group’s solidarity, its sense of its moral worth, its traditions, its belief in the value of its contributions to the society, seem to have lost status and respectability, *alienation* from the society as a whole or from specific elements of it viewed as gaining greater and perhaps undeserved recognition, becomes a likely consequence.

This dynamic of alienation has become increasingly apparent with the last several decades of social change in the American societal community. In decades after War II, many groups in the society gained new participation in statuses of the respectable middle class. Factory workers in many industries, often supported by unions, entered the middle class from statuses that, as “working class”, experienced exclusion from the middle class in pre-war social life. Public servants, such as police officers, firemen (there were no firewomen at the time), sanitation workers, and clerical staff in offices also advanced into the middle class Aspects of these changes in status were due to improved economic circumstances and the resulting changes in life conditions, importantly home-owning, but also to advances in recognition of the importance of their social roles. The middle class became much less exclusive, hence a much larger proportion of the society. Concomitantly, exclusion from the middle class came to involve a deeper sense of social deprivation, especially for groups having the experience of falling out of the middle-class. The deprivation also gained a political dimension in that neither major political party and only few candidates for political office campaigned to advance the interests of citizens excluded from the middle class; political appeals were to advance the interests of members of the middle class, now a large majority of voters.

To understand the current crisis of American society, it is essential to focus on divisions that have emerged regarding the statuses and levels of respectability accorded to certain social groups. Economic changes have introduced important divisions. Ever larger and publicly more prominent groups of extremely wealthy individuals—the billionaires and multi-millionaires—have emerged from the financial, banking, and technology fields. Their activities and extraordinary prosperity have been widely reported in the mass media. Many lawyers and some physicians in specific specialties at major medical centers have also gained great wealth. High executives in large corporations have prospered—with salaries, bonuses, and shares of stock often amounting to tens of millions of dollars per year. Yet, other sectors of the economy have languished. Small farmers are often hard pressed to make sufficient profits to sustain a middle-class style of life. Large industrial farms can be highly profitable for owners or investors, but they employ large numbers of farm workers who receive low pay and generally live under poor conditions. The closing of factories in many industries in the “rust belt” cities have reduced families to less remunerative work and poorer lifestyles, often limited to declining neighborhoods. Many rural areas, villages, and small towns have found their bases of livelihood and social status seemingly marginalized by the broader society. For decades, many children of these communities have moved to metropolitan areas for education and employment, interrupting the continuity of generations. Residents rarely see their ways of life depicted in movies, on television, or discussed in respectful terms in the mass media. For people who live in the Midwest, there is frequent discussion of living in “fly-over country”.

Another dimension of cleavage in the societal community involves education. Polling has shown for years that on a variety of social and political issues there are sharp differences between the college educated and those without college degrees and especially those without some exposure to college education. To some degree, this line of difference overlaps with the line between the middle-class and the working-class, thus different communities of residence and lifestyles, and the line between mainline and evangelical church members. However, the better educated tend to hold more liberal sensitivities and moral outlooks, and, in the politics of the last several years, opposed more firmly to Donald Trump.

These changes have been cumulating over several decades, and they have brought a variety of groups to living under straightened circumstances and experiencing an acute sense of loss of status and respect for their work, ways of life, traditions, and moral sensibilities. These feelings have generated resentment toward the newly wealthy and ultra-wealthy, the urban elites, and the educated groups said to be influential in guiding processes of social change.

The divisions in orientation toward the changes in the status order have been amplified by the degree to which the citizens who have been losing status are also more often fundamentalist in religious orientation. The fundamentalism has brought with it convictions that an older, more traditional status order was legitimate and that its adherents have lost statuses of respect illegitimately. At the same time, the legitimacy of the ways of life of the coastal urbanites, the financiers, the corporate wealthy, the lawyers who protect the wealthy, the heads of large corporations, and so forth are deemed to be questionable. The decency and value of the secular orientations are especially suspect.

Race relations have fed into these patterns of division. For many whites, the older status order, especially as experienced in the working class and lower status-levels of the middle class, included a dimension of racial advantage. Just how that hierarchy has been symbolized in relations among individuals of different races has varied greatly across the country. In the rural South, it included personal deference on the part of African Americans, as in addressing whites as Mr. or Mrs.when they addressed one by first name or even a demeaning nickname. In the urban areas of the North, race relations often involved just social distance, as in living in segregated neighborhoods, working in different and well separated branches of a corporation, shopping in different stores, receiving poor public services,^2^ and sending children to segregated and under-resourced schools. Even today, most urban African Americans continue to live in racially segregated neighborhoods. While this is true especially for the poor and economically marginal, who live in “ghettos”, it is increasingly the experience of the middle class as well. Their residences, too, tend to be segmented off from their social class equals. However, the core of the Black Lives Matter movement is the moral claim of African American citizens to be treated with equality, accorded status, and shown the same respect and civility as whites, and have the opportunity for fellowship with the whites who have made comparable social contributions. Education, work productivity, law-abidingness, decency in style of life, generosity to others, and so forth should bring African Americans the same opportunities and social statuses as they do whites.

The moral claims of African Americans tend to invoke the sharpest opposition among whites who have experienced declines in status for their lifestyles. The Black Lives Matter movement has threatened especially the whites who feel most acutely that the status order of several decades ago was more legitimate, even religiously ordained. While they may accept changes in interpersonal relationships among the races, they nevertheless are uncomfortable with broader changes affecting positions in society now advocated by supporters of Black Lives Matter, including the many whites who joined the summer demonstrations. The sense of threat is further heightened by the demonstrations focusing on the use of public authority by police, the courts, the system of incarceration. The sense of threat is heightened when, as was common in the summer of 2020, the advocacy for change of Black Lives Matter, was supported by elected officials. In addition, many whites fear the time, only a couple of decades in the future, when they will become a minority in the nation’s population and may then lose more economic advantage, political power, and prestige.

Although the nation’s focus over the past several months has been on the claims of African Americans, the statuses of other groups are also at issue. In the Southwest and in California, the numbers of Latino Americans, some of whom are recent immigrants, others of whom have lived in the U.S. for generations, are greater than African Americans. Latinx have suffered social exclusions somewhat comparable to African Americans, so the Black Lives Matter movement has raised issues of justice and fairness for them as well. They, too, are articulating moral claims against discrimination and are asserting their right to social equality. Asian Americans, a diverse group in terms of cultural backgrounds, yet often viewed as unitary in the society at large, have received less discrimination, but have ample reasons to assert their own claims to a new equality. South Asian Americans, mostly from India, but many from Pakistan, have often come to the U.S. with greater economic skills and sometimes wealth, hence may experience less discrimination. Yet, as people of color, they have frequently identified with the movement of racial equality. As immigrants, they have often experienced the reaction of whites and on occasion other groups that they are taking jobs away from longer-established Americans, thus activating the reaction that the America of several decades ago was a better society without them.

The Black Lives Matter demonstrations have generated positive changes. Many corporations have committed to pursuing new policies of “diversity” in hiring and promotions, as have many non-profit institutions, including universities. Newspapers, television and cable news and commentary shows are including African American and Latinx commentators. Cities are revising policing practices. The real estate industry may be ending some of its restrictive and segregating practices. In 2020, more African Americans gained election to public office, perhaps best symbolized by the Warnock victory in Georgia. Although these changes indicate the possibility of a better future, it is also apparent that many whites have reacted to the new claims of non-white groups to equal justice and greater economic opportunity in terms of a nostalgia for a previous social order. Such nostalgia has been especially strong among religious fundamentalists and among citizens who feel they have lost economic well-being and among groups that have been experiencing decline in social status and respect. The same groups provided much of Trump’s enthusiastic “base” in the 2020 election, a base strengthened by reactions to the Black Lives Matter movement and a center for recruitment into the Proud Boys, Oath Keepers, and other militias. Initial reports that many of the militia members who attacked the Capitol on January 6 were small business owners, not workers in displaced industries, were later supplemented by information showing that many of them had in fact lost their businesses or were on the verge of losing them due to pandemic “lockdowns”.

The pandemic produced some important changes in qualities of social relationships. A major factor was the imposition of social isolation in degrees that have varied during the year and from placed to place. Many families have experienced substantial confinement to the household and thus loss of interpersonal connection with members living independently and with other relatives. Friendships and neighborly relationships have been similarly dislocated. Where individuals have been able to work from home, they have lost day-to-day connection with colleagues or had such connection attenuated to Zoom meetings. Where younger children are attended school virtually by Zoom, parents have had to give up other activities, including out-of-the-home jobs for many mothers, to supervise them. Daily or weekly rounds of shopping have been disrupted, along with the interpersonal connections they often involve. People who have lost employment have become dependent on unemployment compensation, SNAP benefits, and deferral of rent or mortgage payments. Many who had believed themselves in the solid middle-class have had to join long lines of cars waiting for food distribution.

The disruptions to informal community ties have precipitated a variety of harms. Rates of personal depression have increased, as have feelings of trauma and uncertainty. Worries that career paths have been disrupted are common among the young, including recent high school and college graduates. Rates of substance abuse have increased, especially for alcohol and for opioids and other sedating drugs. Cities report increasing numbers of deaths due to heroin and fentanyl overdoses. Rates of domestic abuse and violence have increased. Although rates of thefts have apparently not increased, many cities have experienced dramatic increases in shootings and gun-related deaths. Many families, especially in slum neighborhoods, have reduced the frequency with which they go out for walks and have restricted children from outdoor play.

Social division has also emerged over measures to contain the Covid pandemic. Instances of arguments and fights over masking have been reported in the news. Where states or cities have closed or restricted attendance at bars, restaurants, gyms, and shops or have resisted such measures, political and interpersonal dispute and division have resulted. President Trump supported resistance to “lockdown” measures and criticized the epidemiologists and infectious disease specialists who advocated for them, undermining confidence in pertinent science. Most divisively, he responded “Liberate Michigan” after the governor of that state instituted “lockdowns” and refused to criticize a militia that planned to kidnap her. Even though Trump bragged about the support his administration gave to vaccine development, he also seemed to support vaccine skeptics. In these respects, questions of Covid policy have divided groups skeptical about the pandemic or scientific measures to contain it from the mainstream.

The economic decline that resulted from efforts to control the Covid pandemic also had significant consequences for the societal community. An important factor was the additional inequality that resulted. The stock market rose, driven largely by stocks in finance and “tech”, such as Twitter, Facebook, Google, and Zoom, that were used more heavily by people isolated at home. Many of the wealthy benefitted during a year when many others lost jobs and suffered the insecurities of lack of income. As people lost jobs, their inability to pay rent or make mortgage payments passed economic pressure to landlords and banks. Legislation banning evictions helped families that could not make payments over the short term, but left them fearful about their ability to make back payments when the bans would be lifted. The legislation thus sustained uncertainties both for tenants and homeowners and for landlords and banks into the future. Difficulties in finding funds to cover routine expenditures, from food to college tuition, strained relationships in families, but also relations of families to neighbors, shops, service providers, and others. Shopkeepers, restaurant and bar owners, and small contractors experienced their businesses failing or at risk of failing, sometimes after decades of having built them to prosperity. The sense of loss was not only economic; it extended to sense of status, contribution to the community, and pride.

Exacerbating the divisions in the community were differences among the “voices” of the mass media and social media. Although these differences had been mounting for several years, amplified by the divisive role of Donald Trump, they grew more severe in the course of 2020. People who watched Fox News and people who watched CNN or MSNBC, those who identified with the political right versus the political left, aligned differently on a wide range of issues. Similarly, 6.5 million citizens who read the New York Times, whether in print or online, and those who read the Washington Post, tend to have markedly different understandings of national affairs than citizens who draw their news from the Murdock family media, Fox News, the New York Post, or The Wall Street Journal. In using social media, people tend to join – or are steered by algorithms—to groups of like-minded others, further precipitating social divisions. The emergence of QAnon had a similar divisive effect; it sustained and amplified alienated perspectives on national affairs. Trump’s refusal to criticize QAnon and other conspiracy theories amplified their effects. He managed to say both that he did not know enough about them to state a view of them and that, since they supported him, he presumed that they were of value. Followers of QAnon claimed, after being arrested for the attack on the Capitol, that they were surprised to learn that its statements about the stolen election were untrue, thereby showing the importance of differences in information derived from social media as well as mass media. The media-induced cleavages across the societal community contributed to the depth of the political divisions amplified in the course of 2020.

From the time that he became a political figure, Donald Trump had promoted divisions among citizens, as in his advocacy of the “birther” view of the illegitimacy of Obama’s presidency. His blunt “lock her up” attacks on Hillary Clinton and derisive nicknames for opposition politicians, his crude arousal of crowds at his rallies, and policies intended to stigmatize particular groups, such as immigrants crossing the Mexican border, were all designed to be divisive. As the 2020 campaign progressed, his rhetoric and perspective grew more divisive. One index of this is the degree of disrespect that he expressed about Joseph Biden, characterizing him as physically, intellectually, and morally weak and unfit. A result was to divide the citizenry in a manner that Americans have likely not experienced since the Civil War. Republicans who refused to acknowledge that Biden had legitimately won the presidency even weeks after he had taken office were sustaining a serious cleavage. Moreover, Trump and many Republican members of Congress continued to articulate the idea that the nation had illegitimate leadership and administration for over a month after Biden’s inauguration. After Trump’s second impeachment trial failed to convict him, and after the devastating information about his role in the attack on the Capitol presented by the House managers, most Republican members of Congress and state party leaders maintained their support for Trump’s leadership. Many said that they looked forward to his 2024 candidacy for the presidency, despite the margin by which he had lost the 2020 election and the legal risks he faces from both civil and, likely, criminal suits. Their loyalty to Trump, based on their confidence in the support of his “base” and their rejection of other groups of citizens expressed a profound division within the societal community.

In early 2021, it was apparent that the nation’s citizenry – its societal community – was complexly divided. Although some commentators spoke of citizens acting politically out of “tribal” allegiances, in fact the Democratic and Republican alliances are composed of a variety of social elements. Members of each party-centered alliance differ in origin by religious and ideological groupings, regional and local constituencies, educational status, occupational and professional roles, and family political heritages, to cite some key elements. However, what became dramatically apparent on January 6, is that many thousands of citizens were so deeply alienated from Constitutional and legal institutions that they believed Trump’s “Stop the Steal” claims about the election despite its rejection by 60 courts, by certifying officials in each of the “battleground” states, and by properly certified votes, showing that Trump had lost by 7 million votes, by affirmation of the election outcome in the Electoral College, and by reporting in the major newspapers and television networks, aside from Fox News. Moreover, hundreds, likely more than a thousand individuals, were willing violently to assault the Capital and police protecting it, invade Congressional offices, and seek out members of Congress and the Vice President for planned physical assaults, all blatant violations of law, based on belief in “Stop the Steal”. That such beliefs and violent conduct could occur—and could be encouraged by President Trump and his immediate supporters – represents a profound level of alienation from the societal community and perhaps the gravest crisis of confidence in American society that the nation has faced in over a century and a half.

## Conclusion

The 2020 crisis in American society was complex. It involved division over the Black Lives Matter movement, the Covid pandemic and the losses in lives and health that it induced, the economic recession precipitated by the pandemic and efforts to control it, and the year’s political campaign. Each of these complex events fostered division within the societal community. In early 2021, it seems that the social cleavages are likely to endure, even after the nation makes substantial progress in overcoming the more particular crises of race relations, the pandemic, the recession, and perhaps even the differences induced by the figure of Donald Trump and his leadership of the Republican Party.

It is thus notable that President Biden, in his victory speech and again in his inaugural address, emphasized that he would govern in the interests of all Americans, Trump’s supporters as well as his own. He proclaimed that the country should not be divided between “red” and “blue” but united. In the following days, he proceeded methodically to prepare policies to control the pandemic, revive the economy, overturn many of President Trump’s executive orders, change the direction of the nation’s foreign affairs, and plan a new administration, including appointments to the White House staff, the cabinet, and federal agencies. Vice-President Elect Harris was with him when he made public statements, and she clearly pleased minority populations in her appearances and in delivering her own public statements. Television coverage praised both Biden and Harris in much less reserved terms than during the campaign.

Yet, the national situation remained unclear in important respects. The crises of the pandemic and the economy remained unresolved, even if the pandemic was beginning to moderate by mid-February. Biden was actively addressing the pandemic, the economy, and national divisions from the day he took office, but the pandemic, as predicted by epidemiologists, had soared in numbers of new infections, hospitalizations, and deaths in the weeks beforehand. The crisis of race relations was in practical terms unchanged, but with the hope of policies that would advance toward the goals of Black Lives Matter. The political tensions appeared unremitting, with Republicans in Congress having blocked Trump’s conviction in the impeachment trial and threatening firm opposition to Biden’s bold, $1.9 trillion, relief proposal for the pandemic and economy.

The looming matter before the country in longer term perspective concerns Trump’s “base”: Would it sustain a commitment to Trumpism? Would it remain a solidary political block? Was it mobilizable by other leaders in the Republican Party? Or would it fade with Trump out of office and perhaps preoccupied by both civil and criminal lawsuits? Could the success of Biden’s administration be such that the sense of grievance and disaffection among Trump’s followers would be allayed? The complex crisis is unresolved and, while the focal aspects of it may prove resolvable – they are mostly the kind of practical matters that Americans excel in fixing – the cleavages in the societal community threaten to endure.

## Footnotes

1 My wife and I own a small field of about 14 acres in Otsego County, New York, part of what was once a larger farm in my wife’s family. We rent the field to a local dairy farmer who uses it to grow food for his cows, varying crops from year to year to preserve the quality of the soil, now certified as organic. My wife and I went across the county to Cooperstown about a dozen years ago to hear a report on the prospects for local dairy farming. The committee of farmers reported that the average size of dairy farms in the county was 250–300 head of cows. They visited a farm in Wisconsin that had 3,000 head of cows and saw the greater efficiencies possible on that scale. They then visited a farm in California with 60,000 head of cows. Feed for the cows arrived by boxcar every morning. The milk was sent to a dairy by pipeline! Their report concluded that farms on a scale typical for Otsego County were not competitive. Since then, a third of the farms in the county have closed. A small proportion of the farms have expanded substantially.

2 Over twenty years ago, a colleague and I drove through neighborhoods in North Philadelphia looking for a storefront that we might rent for a project to provide services to drug users at risk of HIV infection. We observed that we could tell the race of people living in a particular neighborhood by how well or poorly the streets were paved. Streets with white residents were well paved, streets with Black residents were ridden with potholes. This proved true even when the streets were only blocks apart.

